# Risk factors in elderly female patients for refracture within two years following surgery for fragility fractures: a prospective observational study

**DOI:** 10.1007/s11657-026-01689-7

**Published:** 2026-03-28

**Authors:** Lili Wang, Baohua Chen, Cuicui Xin, Ying Guo, Yuzhi Song, Huihui Wang

**Affiliations:** Department of Orthopaedics, Shandong Provincial Hospitaln Affiliated to Shandong First Medical University, No. 324, Road Jing Wu Wei Qi, Jinan, Shandong 250021 China

**Keywords:** Fragility fracture, Refracture, Elderly women, Risk factor, Prospective study

## Abstract

**Abstract:**

This prospective study of 784 elderly women found that age, high fall risk, and frailty increase refracture risk after fragility fracture surgery, whereas family support, hemoglobin, and albumin levels are protective, highlighting the need for comprehensive geriatric assessment.

**Background:**

The incidence of fragility fractures is high among elderly women, and the risk of secondary fractures significantly increases after the first fracture, leading to higher mortality rates, complications, and socioeconomic burdens. Identifying risk factors for refracture is crucial for prevention. However, there is currently limited analysis of risk factors for postoperative refracture in elderly women in China.

**Objective:**

The objectives of this study were to investigate the incidence of recurrent fractures within 2 years after surgery for fragility fractures in elderly women and to identify associated risk factors.

**Methods:**

This prospective observational study enrolled 784 elderly female patients undergoing surgery for fragility fractures at three hospitals in Shandong Province, China, between January and December 2022. Data of a total of 33 variables were collected, including demographic characteristics, comorbidities, laboratory indicators, fall risk, frailty status, family support, and medication use. Univariate analysis and binary logistic regression were used to identify factors associated with refracture within 2 years post-surgery.

**Results:**

A total of 53 patients (6.76%) experienced refracture within 2 years post-surgery. Multivariate analysis revealed that advanced age (OR = 1.103), high fall risk (OR = 7.907), and frailty (OR = 1.482) were independent risk factors for postoperative refracture. Conversely, living with family (OR = 0.416), higher hemoglobin levels (OR = 0.975), higher serum albumin levels (OR = 0.895), and stronger family support (OR = 0.891) were protective factors against refracture.

**Conclusions:**

Preventing postoperative refracture in elderly women with fragility fractures requires a systematic approach. Clinical practice should incorporate comprehensive geriatric assessments, routinely including fall risk evaluation, frailty screening, and nutritional status assessment, while emphasizing and leveraging family support systems. It is recommended to integrate fall prevention education, frailty intervention, nutritional support, and family involvement into standard fracture liaison services, forming multidimensional, individualized management strategies to effectively reduce refracture risk.

## Introduction

Osteoporotic fragile fractures occur due to reduced bone mineral density and/or deterioration of bone microarchitecture, typically as a result of minimal trauma [1]. The World Health Organization (WHO) quantifies this force as equivalent to a fall from standing height or less [2]. Common sites of fragility fractures include the vertebrae, distal radius, hip, proximal humerus, and pelvis [3]. Osteoporotic fragility fractures represent a major health concern among the elderly, characterized by high rates of disability and mortality, and impose a significant economic burden on society [4, 5]. For patients who have already experienced a fragility fracture, preventing subsequent fractures has become the foremost challenge in osteoporosis management. Research indicates that the period following an initial fracture, particularly within 2 years post-surgery, represents a “high-risk period” for subsequent fractures [6, 7]. Compared to the initial fracture, recurrent fractures further exacerbate functional decline, medical costs, and mortality risk in patients [8, 9].

Older women are a high-risk group for osteoporosis and fragility fractures [10]. Although surgery can effectively repair trauma after a fracture, patients still face an extremely high risk of refracture [11]. Currently, several fracture risk prediction tools, such as FRAX [12], are available internationally. However, these models were developed based on Western populations and do not fully account for the comprehensive risk factors specific to the postoperative period. Their applicability to the postoperative elderly population in China remains to be validated. Furthermore, although a nationwide cross-sectional study by Tang et al. [13] identified multiple risk factors for refracture among Chinese women over 60 with osteoporotic fractures, the timing of refractures was not specified, preventing analysis of “recent refracture” characteristics. A review indicates that family caregivers play an indispensable role in managing bone health among elderly patients with osteoporosis [14]. To date, no studies have incorporated family support into analyses of risk factors for refracture following fragility fractures.

Therefore, this study aims to analyze the incidence of recurrent fractures within 2 years post-operatively for fragility fractures in elderly Chinese women, along with associated risk factors, through a prospective observational study. Notably, this research is the first to incorporate geriatric conditions, such as frailty, nutrition, and social support. The findings are intended to provide evidence for clinical identification of high-risk factors and the development of targeted intervention strategies.

## Materials and methods

### Study design

This study adopted a prospective observational design to investigate the incidence and risk factors for refracture in elderly female patients within 2 years following surgery for fragility fractures. The calculation of the sample size was based on the number of risk factors for refracture in elderly female patients within 2 years after surgery for fragility fractures. Through literature analysis, this study included a total of 33 variables. According to the sample size calculation formula [15], the sample size should be 5 to 10 times the number of variables studied, with an additional 20% to account for participant attrition. It was calculated that a minimum of 413 cases ((33 × 10)/0.8)) were required for this study. Finally, a total of 784 cases were included in this study.

### Participants

This study employed a prospective cohort design to select participants. Using convenience sampling, elderly patients with fragility fractures who sought treatment at three hospitals, i.e., the Trauma Center of Shandong Provincial Hospital Affiliated to Shandong Medical University, Jinan Fifth People’s Hospital, and Weifang Yidu Central Hospital Trauma Center between January and December 2022, were enrolled as study subjects. Inclusion criteria were as follows: female; age ≥ 60 years with fracture diagnosis confirmed by X-ray plain film or CT; primary fragile fracture meeting the diagnostic definition of fragile fracture [16]; those undergoing surgical treatment; and those volunteering to participate in this study. Exclusion criteria were as follows: pathologic fracture; death during follow-up; refusal of follow-up; data missing >15%; lost to follow-up; re-admission due to nonunion.

### Ethical consideration

The study protocol was approved by the Ethics Committee of Provincial Hospital affiliated to Shandong First Medical University (SWYX: NO. 2021–485). Written informed consent was obtained from all participants or their guardians. Personal data were encrypted and deidentified in the data analysis process to ensure privacy protection, and the data were used for research purposes only.

### Measurement and variables

1. Risk factors for refracture within 2 years after surgery for elderly fragility fracture. Relevant domestic and international literature [17–19] was compiled and used for screening factors associated with geriatric fragility fractures and developing a risk factor extraction table. This included a general information survey form covering the following information: demographic characteristics (age, body mass index, fracture location, smoking history, alcohol consumption history, place of residence, and inhabiting information defined as living alone or cohabiting with others, primary mode of mobility), medical history (≥ 2 comorbidities, cerebrovascular disease (stroke, cerebral infarction, and transient ischemic attack), cardiovascular disease (hypertension, coronary heart disease, myocardial infarction, arrhythmia, and peripheral arterial occlusive disease), chronic obstructive pulmonary disease (COPD), anemia (hemoglobin < 110 g/L), Alzheimer’s disease (AD) diagnosed according to the Diagnostic and Statistical Manual of Mental Disorders, Fifth Edition (DSM-5), published by the American Psychiatric Association [20], Parkinson’s disease (PD) diagnosed according to the 2015 Movement Disorder Society (MDS) criteria and the Chinese Parkinson’s Disease Diagnosis Criteria (2016 Edition) [21], diabetes, vision-impairing ocular diseases (previously diagnosed conditions including but not limited to cataracts, age-related macular degeneration, glaucoma, diabetic retinopathy, corneal opacity, optic atrophy), and rheumatoid arthritis (RA)), laboratory results (hemoglobin (Hb), serum albumin, serum alkaline phosphatase, serum creatinine, serum calcium, serum phosphorus, blood glucose, and absolute lymphocyte count), and a follow-up questionnaire (postoperative bed rest duration, occurrence of refracture, refracture site, cause of refracture, implementation of anti-slip measures defined as wearing non-slip shoes or installing non-slip mats in bathrooms, postoperative exercise regimen (including at least twice-weekly activities such as walking, jogging, Tai Chi, yoga, dancing, and table tennis), duration of osteoporosis medication use (calcium supplements, calcitriol, bisphosphonates, etc.)).
2. Perceived Social Support from Family Scale. The Perceived Social Support from Family Scale (PSS-Fa) was compiled according to Procidano et al. and Zhang et al. [22, 23]. There were 15 items on the scale, which were scored by two points, i.e., 1 = Yes and 0 = No; higher scores indicated higher family support. Studies have shown that the scale had high reliability and validity [24].
3. Tilburg frailty indicator. Based on Zamora-Sánchez et al.[25] the Tilburg frailty indicator (TFI) involved 15 entries concerning physical, psychological, and social frailty, and the binary “yes” or “no” scoring system was applied. Scores of five points or higher were defined as indicative of frailty, with higher scores corresponding to a more severe degree of frailty.
4. Chinese Version Morse Fall Scale. The Chinese Version Morse Fall Scale was used to assess a patient’s risk of falling by evaluating the presence or absence of various risk factors [26]. This scale comprised six items: fall history, secondary diagnosis, use of ambulatory aids, intravenous therapy or heparin cap use, gait, and cognitive status related to gait. Each item was scored, with a maximum total score of 125 points for all six items. The total score ranged from 0 to 125 points. A score greater than 45 indicated a high risk of falling, a score in the range of 25–45 points indicated a moderate risk, and a score less than 25 points indicated a low risk. A higher score indicated a greater risk of falling. The fall risk assessment using the Chinese Version Morse Fall Scale was performed at the time of admission of the participant, every 7 days during hospitalization, and when the patient’s condition changed. The results of the fall risk assessments were included in electronic medical records.

### Data collection and follow-up

A research team comprising seven members was established, including one associate chief physician, two associate chief nurses, and four graduate students. Prior to data extraction, investigators underwent standardized, homogeneous training and were only permitted to participate in this study after passing an assessment. Specially trained nurses conducted patient assessments, while investigators reviewed medical records or verified details by phone when uncertainties arose. To ensure data accuracy, two individuals cross-checked the data before each independently entered the data into the electronic records. Demographic characteristics and histories of underlying conditions were collected within 24 h of admission. Final laboratory results, discharge fall risk assessment, frailty scores, and family support scores were collected within 24 h prior to discharge. Follow-up periods commenced on the day of discharge for all patients. Telephone or outpatient follow-up was conducted using follow-up questionnaires at 1 month, 6 months, 12 months, and 24 months post-discharge. Follow-up concluded in December 2024. A new fragility fracture was confirmed by imaging. If a patient experienced multiple fragility fractures, then only the earliest occurrence was recorded.

### Statistical analysis

Data analysis was conducted using SPSS 24.0. The normality of the data was assessed using the Kolmogorov–Smirnov test. To compare differences between groups, the chi-square (*χ*^2^) test was used for categorical variables, while the *t*-test or Wilcoxon rank-sum test was employed for continuous variables, depending on normality. Categorical data were presented as numbers and percentages, while normally distributed continuous variables were expressed as mean ± standard deviation (SD). Non-normally distributed continuous variables were expressed as median and interquartile range. Variables with *P* < *0.05* based on the univariate analysis were included in the binary logistic regression analysis, and a forward stepwise regression approach based on maximum likelihood estimation was applied to explore the risk factors for refracture in elderly female patients within 2 years following surgery for fragility fractures. A two-sided *P* < *0.05* was considered statistically significant.

## Results

### Characteristics of participants

**Fig. 1:**
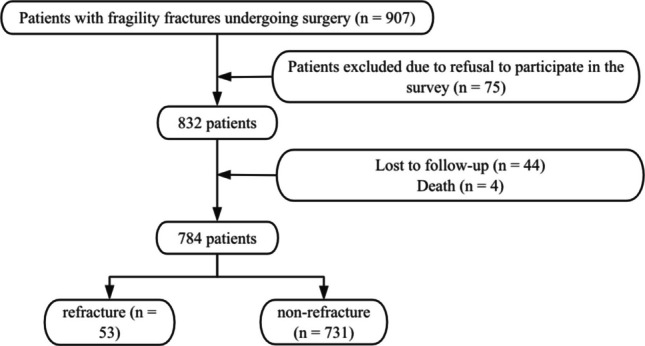
Patient selection procedure in this study

Between January and December 2022, a total of 907 elderly women patients underwent surgical treatment for fragile fractures at the three target hospitals. Following a rigorous screening process, the cohort comprised 784 patients, with 53 experiencing refracture. Among the 123 patients excluded, 75 refused to participate in the survey, 44 were lost to follow-up, and four died during the follow-up period (Fig. 1). Finally, a total of 784 patients were included in this study, with ages spanning from 60 to 102 years old (a median of 74.0 (67.0, 81.0) years old) and BMI ranging from 15.70 to 38.65 kg/m^2^ (a median of 23.81 (21.67, 26.75) kg/m^2^).

### Univariate analysis of refracture in elderly women with fragility fractures within 2 years after surgery

The Wilcoxon test and the chi-square test showed no statistically significant differences in residence, bed rest days, exercise, drink, smoke, BMI, drug time, anti-skid, complication, cerebroVD, cardioVD, COPD, anemia, AD, diabetes, eye diseases, RA, creatinine, calcium, phosphorus, glucose, and lymphocyte between the refracture group and no-refracture group (*P* > 0.05). However, age, inhabiting information, fracture site, walk mode, fall risk, Parkinson, Hb, albumin, aLP, frailty, and support showed statistically significant differences (*P* < 0.05) (Table1).

**Table 1:** Univariate analysis of the association between refractures within 2 years of post-operative treatment for fragility fractures and surveyed factors in elderly women (*N* = 784)

Factor	Refracture (*N* = 53)	No refracture (*N* = 731)	*χ*^*2*^/*Z*	*P*
Age	78 (74, 88)	73 (67, 81)	− 5.039	< 0.001
Residence				
Rural aera	20 (37.70%)	267 (36.50%)	0.730	0.694
Town	8 (15.10%)	85 (11.60%)		
City	25 (47.20%)	379 (51.80%)		
Inhabiting information				
Living alone	20 (37.70%)	166 (22.70%)	6.167	0.013
Cohabiting with others	33 (62.30%)	565 (77.30%)		
Fracture site				
Hip	29 (54.70%)	229 (31.30%)	14.587	0.002
Upper limbs	6 (11.30%)	84 (11.50%)		
Lower limbs	7 (13.20%)	95 (13.00%)		
Thoracolumbar spine	11 (20.80%)	323 (44.20%)		
Bed rest days	3 (2.00, 6.00%)	3 (2.00, 3.00%)	− 1.485	0.138
Exercise				
No	46 (86.80%)	636 (87.00%)	0.002	0.965
Yes	7 (13.20%)	95 (13.00%)		
Walk mode				
Walk alone	41 (77.40%)	628 (85.90%)	6.577	0.037
Bed rest	8 (15.10%)	44 (6.00%)		
Relying on a walker/crutches	4 (7.50%)	59 (8.10%)		
Drink				
No	51 (96.20%)	650 (88.90%)	2.787	0.095
Yes	2 (3.80%)	81 (11.10%)		
Smoke				
No	53 (100.00%)	712 (97.40%)	1.412	0.235
Yes	0 (0.00%)	19 (2.60%)		
BMI (kg/m^2^)	23.08 (20.15, 25.25)	23.97 (21.67, 26.76)	− 1.758	0.079
Drug time				
< 1 month	24 (45.30%)	422 (57.70%)	3.545	0.170
1 ~ 3 month	25 (47.20%)	253 (34.60%)		
No	4 (7.50%)	56 (7.70%)		
Fall risk				
Low	7 (13.20%)	439 (60.10%)	4.221	< 0.001
High	46 (86.80%)	292 (39.90%)		
Anti-skid				
No	44 (83.00%)	600 (82.10%)	0.030	0.863
Yes	9 (17.00%)	131 (17.90%)		
Complication				
No	37 (69.80%)	542 (74.10%)	0.481	0.488
Yes	16 (30.20%)	189 (25.90%)		
CerebroVD				
No	43 (81.10%)	584 (79%)	0.048	0.827
Yes	10 (18.90%)	147 (20.10%)		
CardioVD				
No	26 (49.10%)	344 (45.70%)	0.225	0.635
Yes	27 (50.90%)	397 (54.30%)		
COPD				
No	52 (98.10%)	709 (97.00%)	0.219	0.640
Yes	1 (1.90%)	22 (3.00%)		
Anemia				
No	50 (94.30%)	666 (91.10%)	0.651	0.420
Yes	3 (5.70%)	65 (8.90%)		
AD				
No	53 (100.00%)	715 (97.80%)	1184	0.276
Yes	0 (0.00%)	16 (2.20%)		
Parkinson				
No	51 (96.20%)	731 (100.00%)	27.655	< 0.001
Yes	2 (3.80%)	0 (0.00%)		
Diabetes				
No	41 (77.40%)	550 (75.20%)	0.120	0.729
Yes	12 (22.60%)	181 (24.80%)		
Eye diseases				
No	53 (100.00%)	712 (97.40%)	1.412	0.235
Yes	0 (0.00%)	19 (2.60%)		
Rheumatoid arthritis				
No	47 (88.70%)	683 (93.40%)	1.742	0.187
Yes	6 (11.30%)	48 (6.60%)		
Hb (g/L)	111 (103.50, 120.00)	120 (109.00, 131.00)	− 4.207	< 0.001
Albumin (g/L)	35.6 (32.05, 38.00)	38.2 (35.00, 40.7-)	− 4.349	< 0.001
ALP (U/L)	68 (61.00, 88.00)	79 (66.00, 98.00)	− 2.39	0.017
Creatinine (µmol/L)	58.66 (51.95, 67.61)	57.85 (50.73, 68.11)	− 0.428	0.669
Calcium (mmol/L)	2.25 (2.15, 2.36)	2.25 (2.17, 2.30)	− 0.336	0.737
Phosphorus (mmol/L)	1.18 (1.03, 1.29)	1.13 (1.01, 1.27)	− 0.822	0.411
Glucose (mmol/L)	5.69 (4.98, 6.86)	5.6 (4.87, 6.89)	− 0.117	0.907
Lymphocyte (10^9^/L)	1.43 (0.86, 2.14)	1.33 (0.92, 1.81)	− 0.781	0.435
Frailty	10 (9.00, 12.00)	4 (3.00, 6.00)	− 8.373	< 0.001
Support	6 (3.00, 9.00)	7 (6.00, 12.00)	− 3.341	0.001

### Precipitating risk factors

The results showed that advanced age (OR = 1.103; 95% CI (1.054, 1.155)), high risk of falls (OR = 7.907; 95% CI (3.034, 20.604)), and frailty (OR = 1.482; 95% CI (1.333, 1.648)) were the independent risk factors for recurrent fractures within 2 years postoperatively among elderly female patients with fragility fractures. Conversely, living with family (OR = 0.416; 95% CI (0.186, 0.927)), Hb (OR = 0.975; 95% CI (0.956, 0.994)), albumin (OR = 0.895; 95% CI (0.815, 0.984)), and family support (OR = 0.891; 95% CI (0.805, 0.988)) were protective factors against secondary fractures within 2 years postoperatively (Table2).

**Table 2:** Multivariate logistic regression analysis predicting refracture

Variable	BETA	SE	Wald	*P*	OR	95% confidence interval	
						Upper limit	Lower limit
Age	0.098	0.023	17.528	< 0.001	1.103	1.054	1.155
Inhabiting information	− 0.878	0.409	4.603	0. 302	0.416	0.186	0.927
Walk mode			9.525	0.009			
Relying on a walker/crutches	—	—	—	—	—	—	—
Walk alone	0.609	0.702	0.753	0.386	1.839	0.464	7.282
Bed rest	2.344	0.888	6.963	0.008	10.422	1.827	59.437
Fall risk	2.068	0.489	17.906	< 0.001	7.907	3.034	20.604
Hb (g/L)	− 0.026	0.01	6.751	0.009	0.975	0.956	0.994
Albumin (g/L)	− 0.111	0.048	5.259	0.022	0.895	0.815	0.984
Frailty	0.394	0.054	52.995	< 0.001	1.482	1.333	1.648
Support	− 0.115	0.052	4.834	0.028	0.891	0.805	0.988

## Discussion

This prospective observational study investigated the incidence and risk factors for refracture within 2 years following surgery for fragility fractures in elderly female patients. Our findings indicate that advanced age, high fall risk, and frailty are independent risk factors for postoperative refracture, while co-residing with family, higher hemoglobin and albumin levels, and stronger family support serve as protective factors.

The incidence of refracture observed in this study aligns with previous reports [8, 9], underscoring the high risk of secondary fractures among elderly patients with fragility fractures. Consistent with the findings of Srithuanthong et al. [27], our study further confirms that advanced age is a significant predictor of refracture, likely attributable to age-related declines in bone mass, muscle strength (sarcopenia), and balance function. A key finding of this study is that high fall risk (OR = 7.907) was the strongest predictor of refracture. This is highly consistent with the systematic review by Egan et al. [28], which identified history of falls as one of the primary risk factors for a second hip fracture. Falls are a significant concern among individuals aged 65 and above, with nearly 30% experiencing at least one fall annually [29]. Meta-analytic evidence indicates that older adults with frailty indicators across mobility, psychological, sensory, and neuromuscular domains have a 33–51% higher likelihood of recurrent falls [30]. Falls remain the leading cause of serious injuries in this age group, often resulting in hip, wrist, humeral, or pelvic fractures [31]. According to a national survey, the majority of fall-related emergency visits among older adults occur at home, particularly in bedrooms (25%), on stairs (22.9%), and in bathrooms (22.7%) [32]. Multifactorial intervention strategies based on systematic clinical risk assessments—including strength and balance training—can reduce fall rates and shift high-risk individuals toward a lower-risk profile [33]. Our model incorporates fall risk assessment at discharge, which may enhance patient awareness and support early adoption of fall-prevention measures. Using the Chinese Version Morse Fall Scale to evaluate patients’ risk of falls at home upon discharge can help clinicians identify high-risk individuals early on and develop targeted fall prevention interventions.

Furthermore, this study is among the first to systematically incorporate the multidimensional concept of frailty into a risk model for refracture. Results indicate that for every 1-point increase in frailty score, the risk of subsequent fracture increases by approximately 1.48 times. Frailty is characterized by a cumulative decline across multiple systems and is driven by physiological, psychological, social, and environmental factors [34, 35]. It is well-established that frailty significantly increases vulnerability to adverse health outcomes, including falls and fractures [36]. In particular, older adults with both frailty and a history of fractures face an almost threefold higher risk of sustaining a hip fracture within the next 2.5 years [37]. Previous studies have demonstrated that frailty effectively predicts the risk of future falls, fracture-related hospitalizations, and mortality among community-dwelling elderly women, with the risk increasing significantly as frailty severity worsens [38]. These results suggest that refracture is not merely an issue of the skeletal system, but also a manifestation of decreased physiological reserve and increased vulnerability of the individual patient. In clinical practice, in addition to anti-osteoporosis treatment, conducting comprehensive geriatric assessments and implementing interventions targeting frailty may have synergistic effects in preventing refractures.

Regarding protective factors, this study has demonstrated that strong family support reduces the risk of refracture. A longitudinal study on hip fracture patients revealed that higher and stable levels of social support over 2 years were associated with improved functional outcomes, increased mental health, enhanced nutritional status, and decreased depressive symptoms [39]. Similarly, greater perceived functional support is associated with enhanced rehabilitation outcomes in elderly hip fracture patients [40]. Patients with strong social support are more likely to adopt positive coping strategies, bolstering self-efficacy and adherence to post-fracture care regimens [41]. Family support may exert its effect through multiple pathways, such as improving medication adherence, encouraging and assisting with regular weight-bearing exercise, enhancing home safety, and providing essential psychological support. Concurrently, this study also identified hemoglobin (≥ 120 g/L) and serum albumin (≥ 38.2 g/L) levels as protective factors against refracture. Serum albumin and hemoglobin, as markers of nutritional status, have been demonstrated to be significantly associated with physical frailty, fall risk, and the incidence of secondary fractures [42–44]. Hypoalbuminemia is associated with general physical weakness, impaired mobility, and reduced ability to prevent or mitigate falls [45]. Therefore, nutritional screening and intervention for elderly fracture patients, addressing anemia and malnutrition, should be considered an integral component of a multimodal management strategy for preventing refractures.

In recent years, numerous studies have explored clinical factors influencing fragility fractures, identifying independent risk factors such as age, height, weight, and lifestyle [46]. Based on these findings, various fracture risk assessment tools have been developed, including FRAX, Garvan, and QFracture. Although multiple risk assessment tools have been developed, differences in geography, ethnicity, and cultural background prevent direct application of risk prediction models to different high-risk populations. For instance, research indicates that FRAX does not incorporate risk factors prevalent in the Chinese osteoporotic population [47], leading to underestimation of fracture risk in Chinese elderly women [48, 49]. Furthermore, studies indicate that the intervention thresholds proposed by FRAX are not suitable for the diagnosis and treatment of osteoporosis-related fractures in China, where domestic scholars have explored intervention thresholds under different circumstances [50, 51]. To date, the appropriate intervention values for the Chinese population remain undetermined.

It is noteworthy that although this study included the duration of osteoporosis medication use as a variable, it did not show a significant association based on the univariate analysis. This could be due to large variations in patient medication adherence, the diversity of drug types, or insufficient follow-up time to observe protective effects. Future research is necessary to meticulously evaluate the impact of different types and durations of anti-osteoporosis therapy on refracture risk.

Notably, the chronic kidney disease (CKD) was not identified as an independent risk factor for recurrent fractures within 2 years postoperatively in elderly women with fragility fractures (*P* > 0.05). This finding contrasts with the retrospective study by Lourenço et al. [52], which reported a significant association between CKD and earlier contralateral hip refracture (< 24 months). The discrepancy may stem from differences in study design and population characteristics between these two studies. Our prospective study exclusively enrolled elderly women, whereas Lourenço et al. included both sexes, which could affect the observed association between CKD and fracture risk. Furthermore, differences in CKD definition, disease severity, and regional clinical practices may also contribute to these inconsistent findings. In our cohort, median serum creatinine levels were within the normal or mildly elevated range, indicating that most participants likely had early-stage CKD, in which skeletal complications and fall risk may not yet be prominent. Nevertheless, bone health in CKD patients—particularly those with moderate to severe disease—should not be overlooked. We recommend that in the future, the renal function assessment should be incorporated into comprehensive geriatric evaluation for fragility fracture patients, and it is necessary to further stratify CKD stages and evaluate their associations with refracture risk in elderly women, eventually clarifying the role of CKD in fracture risk stratification.

## Limitations

This study has several limitations. First, as a multicenter observational study based on convenience sampling, selection bias may exist. Second, although 33 variables were included, other potential influencing factors, such as baseline bone mineral density, vitamin D level, and detailed circumstances of falls, were not included. Additionally, the assessments of family support and frailty primarily relied on patient self-report, which is susceptible to reporting bias. Therefore, future prospective studies or randomized controlled trials with larger sample sizes and longer follow-up periods are needed to further validate these risk factors identified in our study and establish effective risk prediction models.

## Conclusion and implications

In conclusion, preventing refractures after surgery for fragility fractures in elderly women is a systematic endeavor involving multiple aspects. Our findings suggest that healthcare professionals should not only focus on patients’ bone health but also adopt a comprehensive geriatric medicine perspective, routinely performing fall risk assessments, frailty screening, and nutritional status evaluation, and encouraging and utilizing family support systems. It is recommended to integrate fall prevention education, frailty intervention, nutritional support, and family involvement into standard Fracture Liaison Services (FLS) to form a multidimensional, personalized management strategy. This approach is expected to effectively reduce the risk of refracture in this vulnerable population and improve their long-term outcomes and quality of life.

## Data Availability

The data are available from the corresponding author upon reasonable request.
